# A Read-Through Drug Put through Its Paces

**DOI:** 10.1371/journal.pbio.1001458

**Published:** 2013-06-25

**Authors:** Roland G. Roberts

**Affiliations:** Public Library of Science, Cambridge, United Kingdom

An estimated 10% of all human genetic diseases are caused by nonsense mutations. These look like the stop codons that occur naturally at the ends of protein-coding sequences in our genomes, but they arise instead in the middle of the protein code, with potentially devastating consequences. Instead of faithfully trundling along a gene transcript, translating it into a useful protein product, the ribosome stops dead in its tracks, leaving the protein incomplete. If only we could somehow trick the ribosome into ignoring these premature stop signs, surely enough full-length protein could be made to substantially improve the symptoms of unfortunate patients.

That was the vision behind the discovery in 2007 of PTC124, a so-called “read-through” drug. PTC124 (also known as Ataluren) was initially identified via an in vitro screen and efficacy was demonstrated in promoting read-through of mutations that cause Duchenne muscular dystrophy, a severe, lethal, and relatively common genetic disease. Subsequently, however, reports of efficacy for this and other genetic diseases have been patchy, and people in the field have started to ask just how good a drug it is.

**Figure 1:**
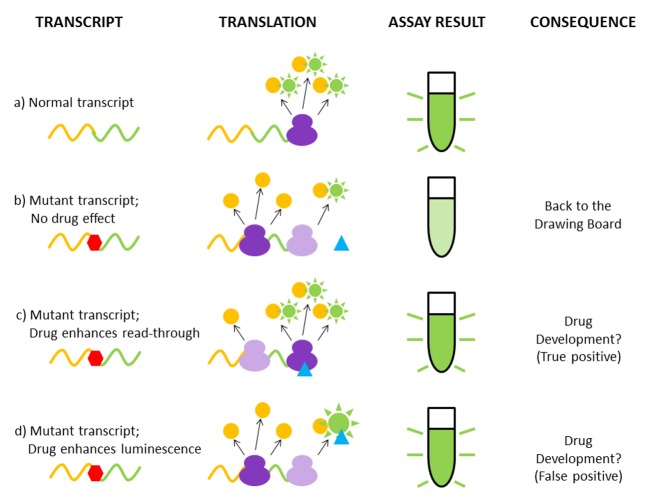
When good assays turn bad. a) A ribosome (dark purple) runs along a normal transcript (wiggly orange/green line), translating it into protein (orange circle – upstream region; green sun – luciferase), which then emits light in the assay. b) A nonsense mutation (red hexagon) stops most of the ribosomes in the middle of the transcript, producing a truncated, non-luminescent protein (orange circle), with very few ribosomes (pale purple) reading through to give a dim luciferase light signal in the assay. The drug (blue triangle) has no effect. c) The dream scenario—a drug interacts with the ribosome, causing it to read through, generating plenty of luciferase and light. d) The potential explanation for the discovery of PTC124—the drug instead interacts with the luciferase molecules, stabilizing them (large green sun) and giving a spurious light signal.

A previous study had hinted at flaws in the assay that was used to screen drugs during the identification of PTC124. The system was based on an engineered transcript, part of which encodes an enzyme from fireflies called luciferase; this can be made to emit light, and is widely used as a “reporter gene” in labs around the globe (Figure 1a). The discoverers of PTC124 introduced a nonsense mutation, just like the ones that cause genetic diseases, so that in the absence of a drug (or in the presence of a drug that doesn't work) very little luciferase was made (Figure 1b). The idea behind this assay is that if you then hit on a drug that actually works, the ribosomes will read through the mutation and generate much more luciferase (Figure 1c).

But what if, instead of tricking the ribosome, the drug tricks the *assay*? What if, say, the drug interacts directly with luciferase and thereby somehow enhances its luminescence, giving the impression of enhanced read-through (Figure 1d)? This seems to be, at least partially, the case for PTC124, which has been shown to bind directly to luciferase and stabilize it.

In this issue of *PLOS Biology*, Stuart McElroy, Irwin McLean, and colleagues fill in the other side of the question: namely, whether PTC124 does nevertheless promote the read-through of nonsense mutations. To do this, they first confirm the spurious effect on firefly luciferase, and demonstrate that this doesn't occur when they instead use a luciferase from an exotic marine animal called the sea pansy, or another traditional reporter gene, beta-galactosidase. They go on to systematically test the effects of PTC124 on the read-through of all possible stop codon contexts (the three-nucleotide stop codon itself, plus the adjacent influential nucleotide) and on a range of different scenarios. In each case, PTC124 fails to enhance read-through, while another well-characterized read-through drug, G418, works to varying degrees.

Where does this leave us with PTC124 and its potential to tackle the 10% of cases of genetic disease that are thought to result from nonsense mutations? We should remember at this point that McElroy and colleagues only test cells (not intact animals), that they only look at the read-through activity of this drug, and that there are several publications that do suggest clinical efficacy (particularly for cystic fibrosis). The conclusion remains, however, that if PTC124 does indeed have beneficial effects on some genetic diseases, it's more likely that this is down to serendipity than the purported mechanism of translational read-through.

Numerous groups are still working on developing effective drugs that can override premature stop codons—in case you wondered, G418 is sadly too toxic for clinical use. PTC124 aside, this manuscript also raises interesting issues for the design and interpretation of high-throughput drug screening assays, and also potentially for the consequences of an initially strong positive screen result on subsequent evaluation of drug efficacy.

**McElroy SP, Nomura T, Torrie LS, Warbrick E, Gartner U, et al. (2013) A Lack of Premature Termination Codon Read Through Efficacy of PTC124 (Ataluren) in a Diverse Array of Reporter Assays. doi:10.1371/journal.pbio.1001593**

